# Let’s post more! The impact of foreign language social grooming on social media on learners’ enjoyment: a moderated mediation model

**DOI:** 10.3389/fpsyg.2025.1674786

**Published:** 2025-10-08

**Authors:** Fei Wang, Xin Wang

**Affiliations:** ^1^School of Humanities, Southwest Jiaotong University, Chengdu, Sichuan, China; ^2^School of Journalism and Communication, China West Normal University, Nanchong, Sichuan, China; ^3^School of Journalism and Communication, Chongqing University, Chongqing, China

**Keywords:** social grooming, foreign language enjoyment, social capital, social support, need for privacy

## Abstract

**Background:**

Digital platforms play an increasing role in shaping family and community language practices, where everyday social grooming may shape the affective side of foreign language learning and maintenance. Yet it remains unclear how such social interaction on social media promotes learners’ foreign enjoyment, through which interpersonal communication processes, and for whom these benefits are realized given differing need for privacy.

**Methods:**

An experiment with random assignment exposed 300 Chinese learners to high versus low intensity social grooming on Chinese social media platforms (e.g., WeChat, Weibo). After 1 week, participants completed validated measures of social capital, social support, enjoyment of language learning, and general need for privacy. A moderated sequential mediation analysis tested the proposed pathways and boundary conditions.

**Results:**

Higher-intensity social grooming increased perceived social capital, which in turn fostered social support; both mechanisms independently and jointly elevated foreign language enjoyment of learners. A stronger need for privacy consistently weakened the direct and indirect effects of social grooming. Diagnostic checks supported the robustness of these patterns.

**Conclusion:**

Social grooming on mainstream platforms can enhance language enjoyment by activating social capital and support, but these benefits depend on learners’ privacy boundaries. By linking social interaction, boundary management, and affective experience, the study advances understanding of how digital practices shape language development and maintenance within the social and affective domain.

## 1 Introduction

In multilingual societies, the widespread use of mobile applications and social media platforms has transformed foreign language learning into a highly visible and interactive social practice. Learners increasingly document their progress publicly, posting vocabulary screenshots, pronunciation clips, and study logs, to share milestones and receive immediate feedback from peers online. While such open visibility can motivate learners by creating a sense of belonging and shared achievement (Nguyen et al., 2024; Zhu and Dawson, 2023), it simultaneously introduces substantial psychological risks, including anxiety, peer comparison, and privacy concerns (Shu, 2023). Empirical research offers mixed evidence. Public exposure on social media may enhance engagement (Jin, 2024; Reinhardt, 2019), or undermine it (Pikhart and Botezat, 2021; Shu, 2023). But increased visibility of social media posts can also trigger anxiety (Wang and Vergeer, 2024), which maybe through peer comparisons. Consequently, the conditions under which social media interactions support positive emotional experiences in language learning remain unclear.

To begin with, positive emotion refers to subjectively experienced feelings with a positive valence, ranging from calm, happy, and satisfied to excited and thrilled (Moskowitz et al., 2021). Experiencing positive emotions, such as those that strengthen social relationships, represents one of the key intervention pathways through which positive psychology seeks to promote well-being (Carr et al., 2021). In this sense, positive emotional experiences on social media can be understood as part of the broader framework of positive psychology, which examines the factors that enable individuals to thrive, achieve well-being, and lead meaningful lives (Csikszentmihalyi and Seligman, 2000; Shen, 2021; Wang et al., 2021). Applied specifically to foreign language learning, this perspective shifts the emphasis from simply reducing negative states such as anxiety (Jin et al., 2021), toward cultivating positive emotions (MacIntyre, 2016; Wang et al., 2021), traits, and environments that sustain engagement and enjoyment.

Scholars in applied linguistics have shown how individual attributes, such as creativity (Nosratinia and Zaker, 2014), perseverance (Teimouri et al., 2022), hope (Lei and Lei, 2022), and learner autonomy (Paradowski and Jelińska, 2024), support effective language acquisition. Additionally, interpersonal resources identified by Dewaele and MacIntyre (2016) are critical, including a positive setting (a sense of fulfillment derived from the classroom environment), positive private experiences (personal feelings of satisfaction associated with achieving learning milestones), and a positive atmosphere (pleasant interpersonal relationships with teachers and peers). Viewed together, these insights suggest that routine interactions among language learners on social media platforms can provide opportunities to cultivate and reinforce beneficial emotional and interpersonal resources in line with positive psychology.

Building on this theoretical foundation of positive psychology, social grooming emerges as a specific operational application. Social grooming refers to purposeful, relational self-disclosures and responsive interactions, such as sharing language achievements, posting practice recordings, or exchanging supportive messages, which are intentionally performed to initiate, maintain, and strengthen interpersonal ties (Lin, 2019; Wang and Wang, 2025). From a positive psychology perspective, these deliberate social exchanges enhance learners’ emotional experiences by fostering social capital (the breadth and strength of learners’ social networks) (Gregersen et al., 2016) and social support (the emotional encouragement and practical assistance exchanged within these networks) (Li et al., 2014). Within foreign language learning contexts, these frequent, brief social grooming interactions can transform solitary language practice into shared accomplishments, enhancing learners’ enjoyment, defined here as the positive emotional experience derived from engaging meaningfully and successfully with language learning tasks. Specifically, enriched social capital offers learners diverse linguistic resources and supportive role models, while strengthened social support provides emotional reassurance and instrumental assistance. Together, these interpersonal resources sustain motivation, buffer anxiety, and contribute to ongoing positive experiences with language learning.

Social grooming via social media platforms also brings privacy concerns into sharp focus (Lin et al., 2024; Wang and Wang, 2025). Learners’ online disclosures, such as sharing study logs, vocabulary progress, or pronunciation exercises, may inadvertently reveal sensitive personal information, thereby increasing the risk of boundary violations. Communication Privacy Management theory (Petronio and Child, 2020) explains that individuals actively regulate their informational boundaries according to their personal preferences for openness or privacy. Learners with a high need for privacy (Frener et al., 2024) might manage these boundaries through specific strategies, such as restricting access to certain posts, selectively sharing achievements with trusted peers, or using pseudonyms to minimize identifiability (Wang and Wang, 2025). Conversely, learners with a lower privacy need may adopt more relaxed boundary-management practices, readily sharing their language-learning progress publicly. Consequently, the extent to which social grooming enhances foreign language enjoyment is likely contingent upon learners’ privacy comfort and their chosen strategies for managing personal disclosures and visibility online.

Despite the theoretical clarity, the current literature exhibits several empirical limitations that warrant further study. First, previous scholars focus on Facebook and Twitter (Ariantini et al., 2021; Barrot, 2022), the educational potential of general-purpose social media platforms in Chinese context, such as WeChat and Weibo, remains inadequately examined. These platforms have massive user bases in China and are widely used for everyday communication and content sharing. The key difference is that Chinese platforms integrate many features and mini-programs, unlike Facebook and Twitter. This leads to their high frequency of use. For example, WeChat is with 1.402 billion monthly active users in 2025 (Tencent, 2025), showing its reach as a dominant channel of social interaction. As a result, they function more like super social media platforms. Additionally, these two platforms represent two distinct types of social media: WeChat, a private, closed social media platform focused on intimacy, and Weibo, an open social media platform designed for broadcast-style dissemination. WeChat requires users to choose who can view posts, creating a private and selective experience. Weibo enables broadcast-style or viral dissemination of information. For closed social media, WeChat Moments functions as a key space where individuals document and curate aspects of their daily lives. For open social media, Weibo as serves a similar purpose of public sharing and interaction. Given prior evidence that social relationships are one of the core frameworks for foreign language enjoyment (Botes et al., 2021; Dewaele and MacIntyre, 2016), thus, these social media platforms may use digital social relationships to generae foreign language enjoyment in Chinese contexts.

Second, the expansion of digital social relationships may generate a range of social benefits, particularly social capital and social support (Liu and Yeo, 2024). Prior research has usually examined these constructs separately. Studies on social capital include work on competence in multiple foreign languages as cultural capital in family language policy (Xu et al., 2024), the influence of social capital on English language fluency (Wang, 2020), English proficiency as a form of digital social capital (Carolan, 2022), and the role of capital and habitus in English language learning among rural lower-class learners (Wu and Tarc, 2024). In contrast, research on social support has focused on topics such as the role of academic buoyancy and social support in motivating English as a Foreign Language learners in higher education (Jia and Cheng, 2022), the contribution of perceived social support to online language learners’ engagement (Luan et al., 2023), the effect of social support on Chinese English-language learners’ achievement (Wu and Lin, 2023), and the impact of emotion regulation strategies on the relationship between teacher and peer social support and language learning engagement among adolescents (Zhang et al., 2024). Social capital and social support are conceptually distinct but often interconnected (Liu and Yeo, 2024). Social capital provides the structural basis for relationships, while social support reflects the exchanges that sustain learners’ emotional and academic engagement. Considering them together is therefore important for clarifying how different forms of social relationships contribute to learners’ enjoyment in digitally mediated environments.

Third, while social media can strengthen social ties, any self-disclosure entails privacy exposure (Frener et al., 2024). Communication Privacy Management theory highlights boundary regulation in online interaction (Petronio and Child, 2020). Yet, language education research rarely models privacy need as a moderator of instructional or affective outcomes. Reviews of higher-education privacy research show a predominant focus on beliefs and behaviors, often in social media contexts, without linking privacy dispositions to learning emotions such as enjoyment (Kularski and Martin, 2022). However, legal and risk concerns remain salient when grades or feedback are shared in public or semi-public spaces (Chang, 2021), such as social media. Researchers have noted that privacy concerns can reduce social media engagement by prompting protective behaviors, lowering trust, and contributing to fatigue (Bright et al., 2021). In addition, social media can also create low-stakes spaces for identity exploration, connection, and extended participation. These benefits coexist with tensions about protecting students’ data and boundaries (Greenhow and Chapman, 2020). Building on these reasoning, we treat learners’ general need for privacy as a stable boundary condition that can dampen or amplify the social benefits of language-focused social grooming on social media.

We summarize the three limitations of previous researches into three theoretical gaps. First, conceptual compartmentalization remains common. Most studies (Lei and Lei, 2022; Teimouri et al., 2022) treat learner traits (Chen et al., 2022), interpersonal processes (Ding, 2021), and digital environments (Barrot, 2022) as separate predictors, rather than modeling how online relational behavior channels resources into enjoyment. Second, mechanistic under-specification follows from this compartmentalization: although social support (Jia and Cheng, 2022) and social capital (Carolan, 2022) are frequently invoked, few studies test them concurrently as parallel conduits through which a specific behavior, particularly online social grooming (Wang and Wang, 2025), might affect foreign language enjoyment. As a result, the relative importance and interrelationship of these pathways remain unclear. Third, empirical narrowness limits generalizability. Research remains heavily classroom-centered (Wang et al., 2021; Wu and Kabilan, 2025). Few studies examine ubiquitous non-academic platforms such as WeChat and Weibo (Ariantini et al., 2021; Barrot, 2022), and boundary-regulation factors (Frener et al., 2024) are rarely considered.

Addressing these gaps requires an inquiry that situates language-focused social grooming in everyday social media settings, specifies its dual interpersonal mechanisms, and tests need for privacy as a boundary moderator. These elements directly reflect the theoretical foundation outlined earlier: positive psychology highlights the role of positive emotions and interpersonal resources in sustaining well-being and engagement, while social grooming provides a concrete behavioral mechanism through which such resources are generated. Privacy need, in turn, represents an important boundary condition that may amplify or constrain these processes. Taken together, these concepts form the basis for the present investigation. Accordingly, this study focuses on two research questions:

1. To what extent does foreign language social interaction enhance enjoyment of foreign languages?
2. How do interpersonal communication mechanisms influence this effect?

To address these question, the present study examines the impact of social grooming in the domain of foreign language study. We conducted a experiment in which learners were randomly assigned to high or low intensity grooming conditions and subsequently measured on validated scales of social capital, social support, and foreign language enjoyment. We also assessed the participants’ need for privacy to explore its moderating influence on both direct and indirect effects. By applying a moderated sequential mediation model (PROCESS Model 85, Hayes, 2022), we clarify the causal pathways by which online interaction influences learner outcomes and identify the boundary conditions under which these effects.

These research questions are designed to address the gaps identified above. First, by integrating learner traits, interpersonal processes, and digital environments in a single framework, the study responds to the problem of conceptual compartmentalization and examines how online grooming behavior connects social interactions to emotional outcomes. Second, by testing social capital and social support together as both parallel and sequential mediators, the study addresses the issue of mechanistic under-specification and clarifies their relative contributions. Third, by situating the investigation in everyday digital contexts such as WeChat and Weibo and by incorporating privacy need as a boundary moderator, the study extends inquiry beyond classroom settings, improves generalizability, and accounts for boundary-regulation factors often overlooked in prior work. Taken together, these questions aim to advance theoretical understanding of how social grooming enhances enjoyment, extend the Social Grooming Model by testing privacy need as a boundary condition, and highlight practical implications for language learning in digital environments.

## 2 Literature review and hypotheses development

This chapter reviews the theoretical foundations and prior research relevant to the present study, and develops hypotheses regarding the mechanisms through which social grooming on social media influences foreign language enjoyment, through social capital and social support, and how these relations vary with learners’ need for privacy.

### 2.1 Foreign language enjoyment

Foreign language enjoyment has been conceptualized as a broad positive emotion that arises when learners’ psychological needs are met during challenging language-learning activities, a view rooted in the work of Dewaele and MacIntyre (2014) and developed further by Botes et al. (2021). Wu and Kabilan (2025) extend this foundation by emphasizing its manifestation as a specific positive emotion experienced when learners overcome limitations and accomplish difficult tasks in classroom contexts. Introduced as the affirmative counterpart to foreign language classroom anxiety, the construct has progressed from an auxiliary measure to “one of the cornerstones of individual differences research” in applied linguistics (Botes et al., 2021; Dewaele, 2022; Li, 2020). Framed within Fredrickson’s broaden-and-build theory of positive emotion (Fredrickson, 2001) and the wider agenda of positive psychology (Wu and Kabilan, 2025), foreign language enjoyment is posited to expand learners’ cognitive repertoires and facilitate the construction of personal and social resources that help learners achieve their language goals. Current evidence supports this proposition: higher reported enjoyment predicts stronger self-perceived competence (Dewaele and Dewaele, 2017), stronger motivation (Pavelescu, 2019), greater willingness to communicate (Dewaele, 2019; Khajavy et al., 2018), accelerated gains in second language comprehensibility (Saito et al., 2018), lower foreign language classroom anxiety (Li et al., 2023), and superior academic achievement (Li, 2020). Thus, foreign language enjoyment, functions not as a fleeting pleasure (Li et al., 2018) but as a facilitative emotional state that sustains effort and enhances performance across multiple dimensions of language learning outcomes.

The factors influencing foreign language enjoyment are commonly grouped into three broad domains (Wu and Kabilan, 2025). Learner-internal factors include enduring dispositions such as personality traits (e.g., openness, agreeableness: Bensalem et al., 2025; Botes et al., 2024), transient capacities such as emotional intelligence (Li, 2020; Li et al., 2021), and self-evaluations of communicative competence (Jiang and Dewaele, 2019). These constructs capture how learners’ personal resources and mindsets significantly influence their experience of foreign language enjoyment. Pedagogical interactive factors center on the teacher-and-task nexus: teacher friendliness, enthusiasm, and supportiveness (Dewaele and Li, 2021; Li, 2025), instructional style (Zawodniak and Kruk, 2019), task characteristics (Chen, 2023), and topic characteristics (Elahi Shirvan and Talebzadeh, 2018) have also been found to play a vital role in predicting learners’ foreign language enjoyment. Contextual social factors encompass classroom climate (Khajavy et al., 2018; Li, 2025), peer relations, and broader socio-biographical variables such as age, gender, and prior experience (Dewaele, 2022; Jiang and Dewaele, 2019). Across these strands, studies portray foreign language enjoyment as a dynamic construct, fluctuating in response to the interplay of intrapersonal resources, instructional practices, and social environments.

Building on these predictor domains, recent studies have identified teacher appreciation, personal enjoyment, and social enjoyment as the immediate experiential components of foreign language enjoyment (Botes et al., 2021). Teacher appreciation reflects learners’ perception of instructor support and enthusiasm; personal enjoyment captures the intrinsic satisfaction of mastering linguistic challenges; and social enjoyment refers to the pleasure derived from peer interaction and a supportive classroom climate. These components can be seen as broadly consistent with the factors reported by Dewaele and MacIntyre (2016), such as positive classroom climate, private satisfaction, and a generally supportive atmosphere. A common feature of previous studies is that scholars have regarded social relationships as a core component of enjoyment in foreign language learning. Importantly, broad social media spaces add additional channels for these components to operate, exp: public endorsements from teachers migrate appreciation cues online, personalized progress posts heighten self-recognition of achievement, and real-time peer feedback extends social enjoyment beyond classroom walls. Given the established link between higher foreign language enjoyment and improved proficiency indicators such as grades (Li, 2020), as well as evidence that frequent second language use accompanied by positive emotions accelerates skill development (Saito et al., 2018), further investigation into how digital channels specifically promote enjoyment is warranted.

### 2.2 Social grooming in foreign language learning

Social interactions on social media are expanding the social process of foreign language learning. Learners acquire and apply language through daily social interactions (Li and Jeong, 2020) with peers and tutors. Through social interaction, people reflect on what they have learned, integrate new elements of knowledge into their overall systems, and share their achievements and experiences at different levels. Therefore, social media moves language interaction from the classroom into an open, always-on digital space. Learners, teachers, and distant peers now meet in timelines and chat threads rather than the same room; a single post can invite replies from across time zones, enlarging the circle of voices in each learning episode (Reinhardt, 2019). This shift means that the social side of language study, asking questions, showing progress, giving feedback, continues well after class and reaches audiences far wider than the physical class (Akbari et al., 2016; Muftah, 2022). This meaning that these online social interaction may reshape foreign language enjoyment. Because learners can derive foreign language enjoyment from social interactions on social media across three dimensions proposed by Botes et al. (2021). For instance, a friendly emoji and quick note from a teacher lifts teacher appreciation, brief word-remembing-count updates feed personal enjoyment, and encouraging peer comments build social enjoyment. Therefore, social interaction on social media facilitate supportive relationships, identity formation, a sense of belonging and resiliency, the direct contact that occur in the social media post on linguistic social networks cover various language registers (Muftah, 2022).

Social interaction via social media posts becomes more than just a form of expression, it is a deliberate act of social grooming. Social grooming refers to purposeful, relational self-disclosures and responsive interactions, such as sharing language achievements, posting practice recordings, or exchanging supportive messages, which are intentionally performed to initiate, maintain, and strengthen interpersonal ties (Lin, 2019; Wang and Wang, 2025). Key attributes of social grooming include intentionality, where interactions have clear social motives; interactivity, characterized by mutual acknowledgment and feedback; and tie-strength modulation, where the frequency, depth, and tone of interactions reflect relationship closeness (Lin et al., 2024; Lin and Chu, 2021; Liu and Yeo, 2024). In language learning contexts, these social relational attributes (Dewaele and MacIntyre, 2016) manifest through routine social media posts, enabling learners to communicate their learning progress, seek peer support, and enhance relationships within language learning communities.

Social grooming through social media may positively influences learners’ emotional experiences, particularly foreign language enjoyment. Engaging in social interactions provides opportunities (Barrot, 2022) for positive reinforcement from peers and tutors, such as encouraging messages, language-related post sharing (Reinhardt, 2020), affirming comments, and symbolic gestures like emojis, all contributing to higher levels of enjoyment. Learners posting their daily vocabulary logs (Hua et al., 2024) or pronunciation challenges often receive peer recognition, thereby increasing their sense of accomplishment and motivating continued effort (Muftah, 2022). Thus, social grooming may be as a critical mechanism that converts solitary language practice into socially meaningful and enjoyable experiences. Based on this reasoning, we proposed:

Hypothesis 1 (H1): social grooming related to foreign language learning positively affects foreign language enjoyment.

### 2.3 Social ties as mediators

There are two types of social ties discussed in this study: social capital and social support. Social capital refers to resources embedded in one’s social networks, resources that can be accessed or mobilized in purposive actions through ties in the networks (Wang and Wang, 2025). Previous research has shown that social grooming activities, such as sharing progress updates, daily logs, or practice clips, on social media platforms can effectively foster and maintain social connections, thereby increasing social capital (Ellison et al., 2007; Lin, 2019; Lin et al., 2024; Liu and Yeo, 2024). Social capital on social networks provide essential resources such as information, guidance, and support that significantly contribute to students’ academic success, especially for those from underrepresented groups (Mishra, 2020). Through these interactions, learners enhance bonding ties (strong emotional connections providing emotional reassurance) and bridging ties (weaker connections offering diverse, information-rich resources). In the context of foreign language learning (Gregersen et al., 2016; Strobel, 2016), these strengthened social relationships offer expanded linguistic input, diverse role models, and varied informational resources, thus positively influencing learners’ emotional engagement and enjoyment. Drawing from this reasoning, we expected:

Hypothesis 2 (H2): social grooming related to foreign language learning enhances foreign language enjoyment through increased social capital.

Beyond building social capital, social grooming simultaneously facilitates learners’ perceptions of social support. Social support is defined as an exchange of resources between two individuals perceived by the provider or the recipient to be intended to enhance the well-being of the recipient (Liu and Yeo, 2024). Social support derived from peers and communities significantly influences academic outcomes, acting as a buffer against educational stress and anxiety (Mishra, 2020). Prior studies have demonstrated that engaging in social grooming behaviors on social media (Liu and Yeo, 2024), such as exchanging supportive comments (Hayes et al., 2016), sending emojis, or receiving affirmation, can increase the perception and availability of social support (Liu et al., 2018; Yue et al., 2024). For language learners (De Ruiter et al., 2019; Liu et al., 2025; Piechurska-Kuciel, 2017), this emotional reassurance, targeted encouragement, and practical guidance can reduce anxiety, boost motivation, and increase confidence, thereby enhancing their enjoyment of language learning tasks. Thus, we expected:

Hypothesis 3 (H3): social grooming related to foreign language learning enhances foreign language enjoyment through increased social support.

The mechanisms underlying social capital and social support are interconnected. Social capital may serve as an foundation for social support, since social connections represent channels through which supportive interactions occur (Mishra, 2020). Engaging in frequent social grooming helps learners build larger and more diverse social networks, increasing their opportunities to receive social support. Empirical studies further support this pathway (Kalaitzaki et al., 2021; Liu and Yeo, 2024), indicating that broader networks resulting from social media use often precede greater perceptions of available support. In turn, this elevated social support significantly enhances foreign language enjoyment. Thus, social capital may foster social support, creating a sequential mediation effect. Hence, we proposed:

Hypothesis 4 (H4): social grooming related to foreign language learning enhances foreign language enjoyment sequentially through increased social capital followed by increased social support.

### 2.4 Privacy as a boundary condition

Social grooming requires learners to publicly share their progress. As a form of self-disclosure (Wang and Wang, 2025), social grooming may create potential pressures that can hinder some learners. The effectiveness of social grooming thus depends significantly on learners’ privacy preferences as critical boundary conditions. The need for privacy refers to an individual’s stable, cross-situational preference regarding how much access others have to the individual’s self, or to what extent an individual withdraws from social interaction (Frener et al., 2024). We conceptualize this need for privacy as a higher-order psychological preference that does not necessarily cause deficiency or distress if unfulfilled, but rather reflects a strong, consistent individual inclination (Frener et al., 2024). Specifically, the informational need for privacy pertains to individuals’ desire to regulate disclosure of personal information or data to specific audiences (Frener et al., 2024). Psychological need for privacy, on the other hand, concerns individuals’ preferences regarding access to their cognitive inputs, such as attitudes, beliefs, and values, and their deliberate disclosure of cognitive outputs, such as thoughts, feelings, or secrets (Frener et al., 2024). Such privacy management is essential for fostering meaningful interpersonal interactions, constructing self-identity (Altman, 1975), and promoting individual psychological growth (Burgoon, 2012). Therefore, need for privacy represents a persistent motivation to actively manage and regulate personal boundaries, rather than simply responding to particular disclosure situations.

In the context of the Social Grooming Model, social interactions centered around foreign language learning are valuable because they transform minor self-disclosures into meaningful social resources, including social capital and social support (Lin, 2019; Lin et al., 2024). However, every instance of self-disclosure also initiates boundary negotiations described by Communication Privacy Management theory, emphasizing a continuous balancing act between openness and protection of personal boundaries (Petronio and Child, 2020). These two theoretical frameworks intersect clearly in social grooming activities related to foreign language learning, particularly in social media settings. The more frequent and richer these language-related disclosures are (such as sharing vocabulary practice logs or language-learning progress), the higher their potential to generate interpersonal connections and positive emotions; yet simultaneously, these disclosures intensify the need for careful boundary management regarding who can access or share the disclosed content. Boundary-management behaviors (e.g., restricting audiences, lowering disclosure frequency) are situational strategies that express this disposition in specific contexts. Social grooming model therefore treats need for privacy as a moderator of the effects of social grooming, while recognizing that it guides (rather than equals) the moment-to-moment management of disclosure.

Social grooming related to foreign language learning may promote enjoyment via increased social capital and social support, while Communication Privacy Management theory explains the privacy risks associated with these interactions, and the stable individual-level need for privacy determines the default level of boundary control applied by users. Learners with higher privacy needs may employ boundary-management strategies such as restricting their posts to selected audiences, reducing disclosure frequency, using indirect or ambiguous language, or even censoring their own content. Conversely, learners with lower privacy needs may exhibit greater openness, broader audience engagement, and more frequent sharing of personal progress, thus amplifying potential relational benefits. Therefore, social grooming generates interpersonal benefits, communication privacy management theory outlines potential disclosure risks, and the individual’s stable need for privacy moderates the balance of these benefits and risks.

The need for privacy may serve as a moderator between social grooming and its positive effects on foreign language enjoyment, social capital, and social support, functioning as a stable motivational reference point for individuals (Frener et al., 2024). Based on Altman’s (1975) conceptualization of privacy as a balance between desired and actual levels of control, individuals with higher privacy needs tend to establish more restrictive boundary management strategies, carefully selecting both their content and audiences. This heightened privacy management can limit spontaneous engagement and potentially reduce the relational benefits derived from social grooming activities related to foreign language learning. Conversely, individuals with lower privacy needs may tolerate broader visibility and less rigorous content control, thereby more readily benefiting from increased social capital and social support through social grooming. Thus, an individual’s stable need for privacy systematically shapes boundary-management behaviors independent of immediate situational concerns (Dienlin, 2023). Consequently, we expected:

Hypothesis 5 (H5): A higher need for privacy weakens the positive effect of social grooming related to foreign language learning on foreign language enjoymen (h5a), on social capital(h5b) and on social support (h5c)

**FIGURE 1:**
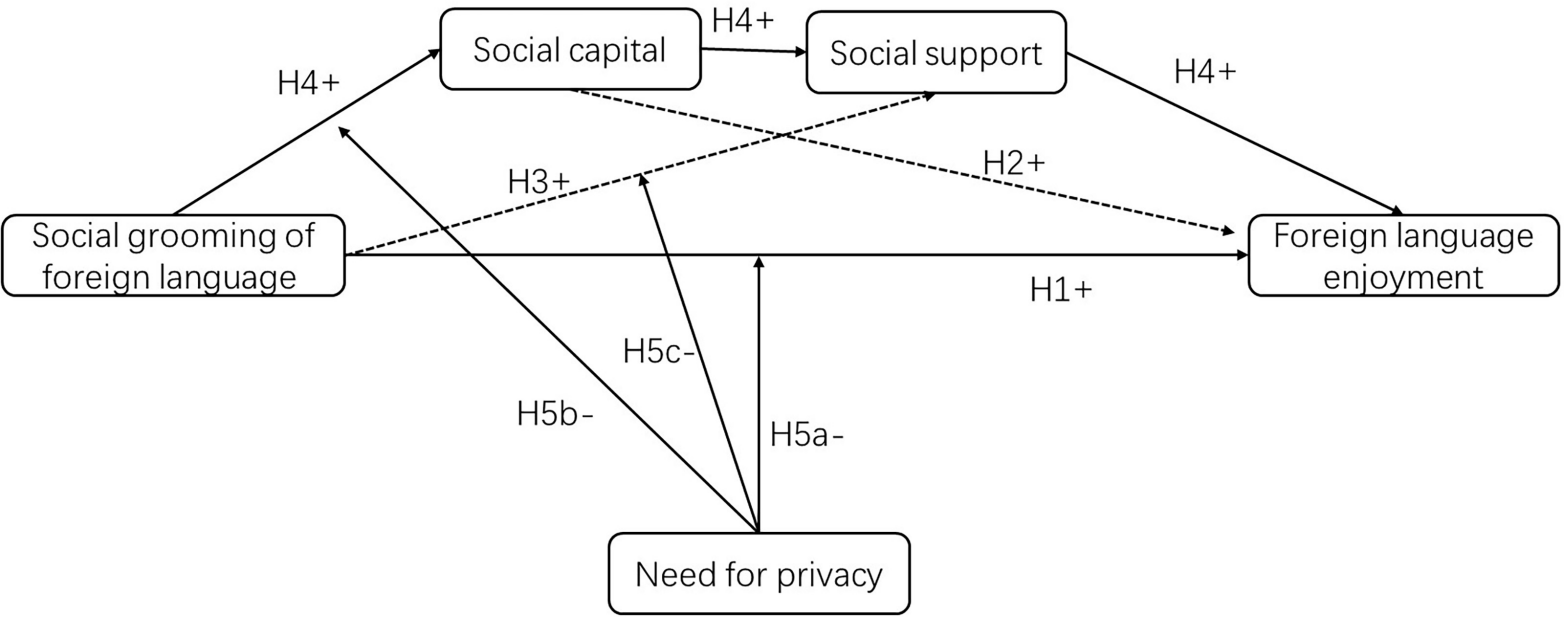
Theoretical model.

Figure 1 visually summarizes the hypothesized moderated mediation model, clearly illustrating the sequential pathways and moderating role of privacy.

## 3 Materials and methods

### 3.1 Participants and procedure

Participants were recruited and the data were collected by Chongqing Rounen Film and Television Media Co., Ltd. (Chongqing, China), a professional media market research company. The experiment was conducted via the company’s platform under the close supervision and design control of the researchers. Ethical approval was obtained from the Academic Ethics Committee of the School of Journalism at Chongqing University (No. xwxy202505001). Participants gave informed consent and were assured confidentiality and voluntary participation. Eligible participants included adult learners (aged 18 to 25 years old, *M* = 21.47, SD = 2.9; 50% male, 50% female) currently learning English as a foreign language, who regularly used social media platforms, specifically WeChat or Weibo, for daily interactions. A total of 300 participants were targeted to achieve a balanced distribution and adequate statistical power, with final recruitment closely matching the targeted demographics. All participants gave informed consent before participation, and confidentiality was assured throughout the study.

Participants were randomly assigned into two experimental groups (high social grooming vs. low social grooming). To ensure clear differentiation between experimental conditions, precise instructions and explicit guidelines regarding expected social grooming behaviors were provided to each participant. Specifically, participants assigned to the high social grooming condition were instructed to actively engage on their chosen social media platform (WeChat or Weibo) for at least seven consecutive days, posting language-learning updates such as vocabulary logs, language progress summaries, or language practice reflections at least three times per day. Participants in this group were also required to regularly interact with peers by responding to classmates’ posts, providing encouragement, feedback, or supportive messages at least three times per day. To ensure that randomization achieved baseline equivalence, we examined age and gender distributions across groups. Adherence to the assigned conditions was monitored via self-reported activity logs (frequency of posts, likes, and comments) completed during the 1-week period. Participants indicated the frequency of posts, likes, and comments they made during the 1-week period. In contrast, participants in the low social grooming condition were instructed to engage minimally, limiting their language-related postings to no more than one per day and interacting only briefly with peers (e.g., liking or providing short comments) no more than once daily.

Before the intervention began, participants completed an initial baseline survey. As part of recruitment, eligibility was screened based on formal English proficiency and educational background. Specifically, participants were required to have either passed the College English Test Band 4 (CET-4), a nationwide standardized examination of English language proficiency administered by the Ministry of Education in China, or achieved a score of 130 or above on the English section of the National College Entrance Examination (Gaokao). Both CET-4 and the Gaokao English section are widely recognized in China as benchmarks of university-level English proficiency. In addition, participants were required to be enrolled at a college or university level or higher. This screening ensured that the sample consisted of learners with a comparable baseline of formal English training, although no baseline language proficiency test was administered within the study itself.

The baseline survey collected demographic information (age, gender, education) and measured participants’ general need for privacy. After the 7-day experimental period, a follow-up survey was administered to assess social capital, social support, and foreign language enjoyment. A manipulation check was also conducted at the conclusion of the intervention to confirm adherence to the assigned intensity of social grooming.

### 3.2 Measurements

Responses will be rated on a five-point Likert scale (1 = strongly disagree, 5 = strongly agree). Participants’ responses were averaged into a composite score, with higher scores indicating higher levels of variable. All variables were measured with good reliability and validity.

#### 3.2.1 Social capital

Social capital (Composite score: *M* = 3.70, SD = 0.45, Cronbach’s alpha = 0.75, KMO = 0.80) was measured using six items drawn and modified from Williams (2006), which has been validated in multiple studies (Liu and Yeo, 2024; Wang and Wang, 2025). Sample items include: “There are several people I trust to help solve my problems,” “There are people with whom I feel comfortable talking about intimate personal problems,” “When I feel lonely, there are several people I can talk to,” “Interacting with people makes me want to try new things,” “Interacting with people makes me interested in what people unlike me are thinking,” and “Talking with people online/offline makes me curious about other places in the world.”

#### 3.2.2 Social support

Social support (Composite score: *M* = 3.39, SD = 0.46, Cronbach’s alpha = 0.88, KMO = 0.92) was measured using the 2-way social support scale drawn and modified from Shakespeare-Finch and Obst (2011), validated in recent studies (Krämer et al., 2021; Xin, 2023). The scale consists of two dimensions: Receiving Emotional Support and Giving Emotional Support. Sample items include: “There is someone I can talk to about the pressures in my life,” “There is at least one person that I can share most things with,” “When I am feeling down there is someone I can lean on,” “There is someone in my life I can get emotional support from,” “There is at least one person that I feel I can trust,” “There is someone in my life that makes me feel worthwhile,” “I feel that I have a circle of people who value me,” “I am there to listen to other’s problems,” “I look for ways to cheer people up when they are feeling down,” “People close to me tell me their fears and worries,” “I give others a sense of comfort in times of need,” and “People confide in me when they have problems.”

#### 3.2.3 Need for privacy

Need for Privacy (Composite score: *M* = 2.99, SD = 0.71, Cronbach’s alpha = 0.94, KMO = 0.96) was measured using eight items from informational and psychological perspective drawn and modified from (Frener et al., 2024). Which has been validated in multiple studies (Dienlin and Metzger, 2024; Wang and Wang, 2025; Zhang et al., 2025). Sample item are: “I would prefer that little is known about me,” “In general, I prefer to remain unknown,” “I do not want my personal data to be publicly accessible,” “Not everyone has to know everything about me”; “There are a lot of things about me that I do not like to talk about with others,” “I feel uncomfortable when others tell me private things about their lives,” “It is hard for me to talk about myself,” “I don’t like it when others talk to me about their private issues.” In this study, need for privacy scale captures an individual-difference disposition; situational boundary strategies are not treated as separate constructs but as behaviors shaped by this disposition.

#### 3.2.4 Foreign language enjoyment

Foreign language enjoyment (Composite score: *M* = 3.64, SD = 0.74, Cronbach’s alpha = 0.95, KMO = 0.95) was measured using eight items drawn and modified from Li et al. (2018), validated in current studies (Li, 2025; Li et al., 2025; Peng and MacIntyre, 2025). Sample items include: “I don’t get bored,” “I enjoy learning English,” “I’ve learnt interesting things,” “I feel proud of my accomplishments,” “It’s a positive environment,” “It’s fun,” “There is a good atmosphere,” and “We form a tight social group.”

#### 3.2.5 Manipulation check

We averaged the five grooming-intensity items into a manipulation-check index (Cronbach’s alpha = 0.81, KMO = 0.85). Participants in the high-intensity condition scored higher (*n* = 145, *M* = 4.60, SD = 0.40) than those in the low-intensity condition (*n* = 155, *M* = 3.00, SD = 0.37), *t* = 14.94, *p* < 0.001, Cohen’s *d* = 1.73, confirming the effectiveness of the manipulation. Sample items include: “During the past week, I share or re-post things regarding foreign language learning on social media (such as WeChat Moment or Weibo)”, “During the past week, I post photos or status updates regarding foreign language learning on social media (such as WeChat Moment or Weibo)”, “During the past week, I see things regarding foreign language learning shared or reposted by friends on social media (such as WeChat Moment or Weibo)”, “During the past week, I see photos or status regarding foreign language learning shared or reposted by friends on social media (such as WeChat Moment or Weibo)”, and “Overall, I interact on social media about foreign-language learning more than usual” (Lin, 2019; Liu and Yeo, 2024; Wang and Wang, 2025).

### 3.3 Analysis

Data analysis involved the SPSS macro PROCESS (Hayes, 2022), specifically Model 85, to test moderated mediation effects. PROCESS Model 85 allows testing the effects of social grooming intensity (high vs. low) on foreign language enjoyment via two parallel mediators (social capital and social support), and moderation effects by the need for privacy on the direct and indirect paths. We ran bootstrap analyses with 5000 bootstrap samples to estimate indirect and conditional indirect effects, providing bias-corrected confidence intervals. Indirect effects were deemed significant if their confidence intervals do not include zero. All variables, except for the independent variable (which is dichotomous), were transformed into z-scores (mean = 0, standard deviation = 1).

As an additional robustness check, all models were re-estimated with age and gender included as covariates. Furthermore, model-fit indices demonstrated that results remained robust and stable in their absence.

## 4 Results

This chapter presents the empirical findings of the study, beginning with baseline equivalence checks and followed by tests of the hypothesized direct, indirect, and moderated mediation effects.

To evaluate baseline equivalence, we compared the two groups on age and gender. An independent-samples t-test showed no significant age difference between the low-grooming (*M* = 21.51, SD = 2.38, *n* = 155) and high-grooming groups (*M* = 21.43, SD = 2.21, *n* = 145), *t*(298) = 0.28, *p* = 0.78, Cohen’s *d* = 0.03. A chi-square test likewise indicated no significant association between group assignment and gender, χ^2^(1, *N* = 300) = 0.01, *p* = 0.91. These findings suggest that randomization successfully balanced participants’ demographic characteristics across conditions.

### 4.1 Direct effect

Hypothesis 1 predicted that social grooming would directly increase foreign language enjoyment. In the outcome model for foreign language enjoyment, the coefficient for social grooming is *b* = 1.20, standard error = 0.07, *t* = 16.60, *p* < 0.001, 95% confidence interval [1.05, 1.34] (Table 1). Hypothesis 1 is supported.

**TABLE 1:** Tested effects.

Path	b	SE	95% CI	p
**Mediation model**				
Social grooming → Social capital	1.05	0.09	[0.87, 1.23]	<0.001
Social grooming → Social support	0.87	0.09	[0.69, 1.04]	<0.001
Social capital → Social support	0.17	0.05	[0.07, 0.26]	<0.001
Social capital → Foreign language enjoyment	0.16	0.04	[0.08, 0.23]	<0.001
Social Support → Foreign language enjoyment	0.14	0.05	[0.05, 0.23]	0.003
**Direct effect**				
Social Grooming → Foreign language enjoyment	1.20	0.07	[1.05, 1.34]	<0.001
**Indirect effects**				
Social grooming → Social capital → Foreign language enjoyment	0.16	0.04	[0.09, 0.25]	–
Social grooming → Social support → Foreign language enjoyment	0.12	0.04	[0.04, 0.20]	–
Social grooming → Social capital → Social support → Foreign language enjoyment	0.02	0.01	[0.01, 0.05]	–
**Total indirect effect**	0.3	0.06	[0.19, 0.41]	–
**Moderated mediation model**				
Social grooming × Need for privacy → Social capital	−0.49	0.09	[−0.68, −0.31]	<0.001
Social grooming × Need for privacy → Social support	−0.75	0.07	[−0.90, −0.60]	<0.001
Social grooming × Need for privacy → Foreign language enjoyment	−0.29	0.06	[−0.41, −0.17]	<0.001
**Conditional direct effect of social grooming (need for privacy)**				
Low (−1 SD)	1.50	0.11	[1.28, 1.72]	<0.001
Mean	1.19	0.07	[1.05, 1.33]	<0.001
High (+1 SD)	0.93	0.07	[0.79, 1.08]	<0.001
**Conditional indirect effect (via social capital)**				
Low (−1 SD)	0.24	0.06	[0.13, 0.37]	–
Mean	0.16	0.04	[0.09, 0.25]	–
High (+1 SD)	0.1	0.03	[0.04, 0.16]	–
**Conditional indirect effect (via social support)**				
Low (−1 SD)	0.22	0.08	[0.08, 0.37]	–
Mean	0.12	0.04	[0.04, 0.20]	–
High (+1 SD)	0.03	0.02	[0.00, 0.06]	–
**Conditional sequential indirect effect (via social capital → social support)**				
Low (−1 SD)	0.04	0.02	[0.01, 0.07]	–
Mean	0.02	0.01	[0.01, 0.05]	–
High (+1 SD)	0.01	0.01	[0.00, 0.03]	–
**Indices of moderated mediation**				
Social capital path	−0.08	0.02	[−0.13, −0.03]	–
Social support path	−0.1	0.04	[−0.18, −0.03]	–
Sequential path	−0.01	0.01	[−0.02, 0.00]	–

### 4.2 Indirect effect

Hypothesis 2 proposed that social grooming enhances foreign language enjoyment through social capital. Social grooming increases social capital (*b* = 1.05, standard error = 0.09, 95% confidence interval [0.87, 1.23], *p* < 0.001), and social capital is positively related to foreign language enjoyment (*b* = 0.16, standard error = 0.04, 95% confidence interval [0.08, 0.23], *p* < 0.001). The bootstrap indirect effect through social capital (evaluated at the median of need for privacy) is 0.16; 95% confidence interval [0.09, 0.25] (Table 1). Hypothesis 2 is supported.

Hypothesis 3 proposed mediation through social support. Social grooming increases social support (*b* = 0.87, standard error = 0.09, 95% confidence interval [0.69, 1.04], *p* < 0.001), and social support is positively related to foreign language enjoyment (*b* = 0.14, standard error = 0.05, 95% confidence interval [0.05, 0.23], *p* = 0.003). The bootstrap indirect effect through social support (at the median of need for privacy) is 0.12; 95% confidence interval [0.04, 0.20] (Table 1). Hypothesis 3 is supported.

Hypothesis 4 predicted a sequential mediating effect (social grooming → social capital → social support → foreign language enjoyment). All component paths are significant (see Table 1), and the serial indirect effect (at the median of need for privacy) is 0.02; 95% confidence interval [0.01, 0.05]. Hypothesis 4 is supported. The total indirect effect (sum of the three paths at the median of need for privacy) is 0.30; 95% confidence interval [0.19, 0.41] (Table 1).

### 4.3 Conditional effects

To test Hypothesis 5, which proposed that learners’ need for privacy would weaken the direct and indirect effects of social grooming on foreign language enjoyment, we examined the interaction of privacy need with social grooming across four pathways: the direct effect on enjoyment (H5a), the indirect effect through social capital (H5b), the indirect effect through social support (H5c), and the sequential path through both mediators.

Consistent with H5a, the interaction between social grooming and need for privacy predicting foreign language enjoyment was negative and significant (*b* = −0.29, standard error = 0.06, 95% confidence interval [−0.41, −0.17], *p* < 0.001). The conditional direct effect of social grooming equaled 1.50 at low need for privacy (−1 standard deviation; standard error = 0.11, 95% confidence interval [1.28, 1.72]), 1.19 at the median (standard error = 0.07, 95% confidence interval [1.05, 1.33]), and 0.93 at high need for privacy (+ 1 standard deviation; standard error = 0.07, 95% confidence interval [0.79, 1.08]) (Table 1). Thus, H5a was supported.

Turning to H5b, the interaction between social grooming and need for privacy predicting social capital was also negative and significant (*b* = −0.49, standard error = 0.09, 95% confidence interval [−0.68, −0.31], *p* < 0.001). The conditional indirect effect via social capital was 0.24 at low need for privacy (95% confidence interval [0.13, 0.37]), 0.16 at the median (95% confidence interval [0.09, 0.25]), and 0.10 at high need for privacy (95% confidence interval [0.04, 0.16]). The index of moderated mediation for this path was −0.08 (bootstrap standard error = 0.02, 95% confidence interval [−0.13, −0.03]) (Table 1). Thus, H5b was supported.

Similarly, H5c predicted that need for privacy would moderate the indirect path through social support, and this expectation was confirmed. The interaction between social grooming and need for privacy predicting social support was negative and significant (*b* = −0.75, standard error = 0.07, 95% confidence interval [−0.90, −0.60], *p* < 0.001). The conditional indirect effect via social support was 0.22 at low need for privacy (95% confidence interval [0.08, 0.37]), 0.12 at the median (95% confidence interval [0.04, 0.20]), and 0.03 at high need for privacy (95% confidence interval [0.00, 0.06]). The index of moderated mediation for this path was −0.10 (bootstrap standard error = 0.04, 95% confidence interval [−0.18, −0.03]) (Table 1). Thus, H5c was supported.

Finally, the sequential pathway linking social capital to social support was also examined. The conditional sequential indirect effect equaled 0.04 at low need for privacy (95% confidence interval [0.01, 0.07]), 0.02 at the median (95% confidence interval [0.01, 0.05]), and 0.01 at high need for privacy (95% confidence interval [0.00, 0.03]). The index of moderated mediation for the sequential path was −0.01 (bootstrap standard error = 0.01, 95% confidence interval [−0.02, 0.00]) (Table 1). Although small in magnitude, this pathway was consistent with the overall hypothesis that privacy need dampens both direct and indirect effects of grooming on enjoyment.

**FIGURE 2:**
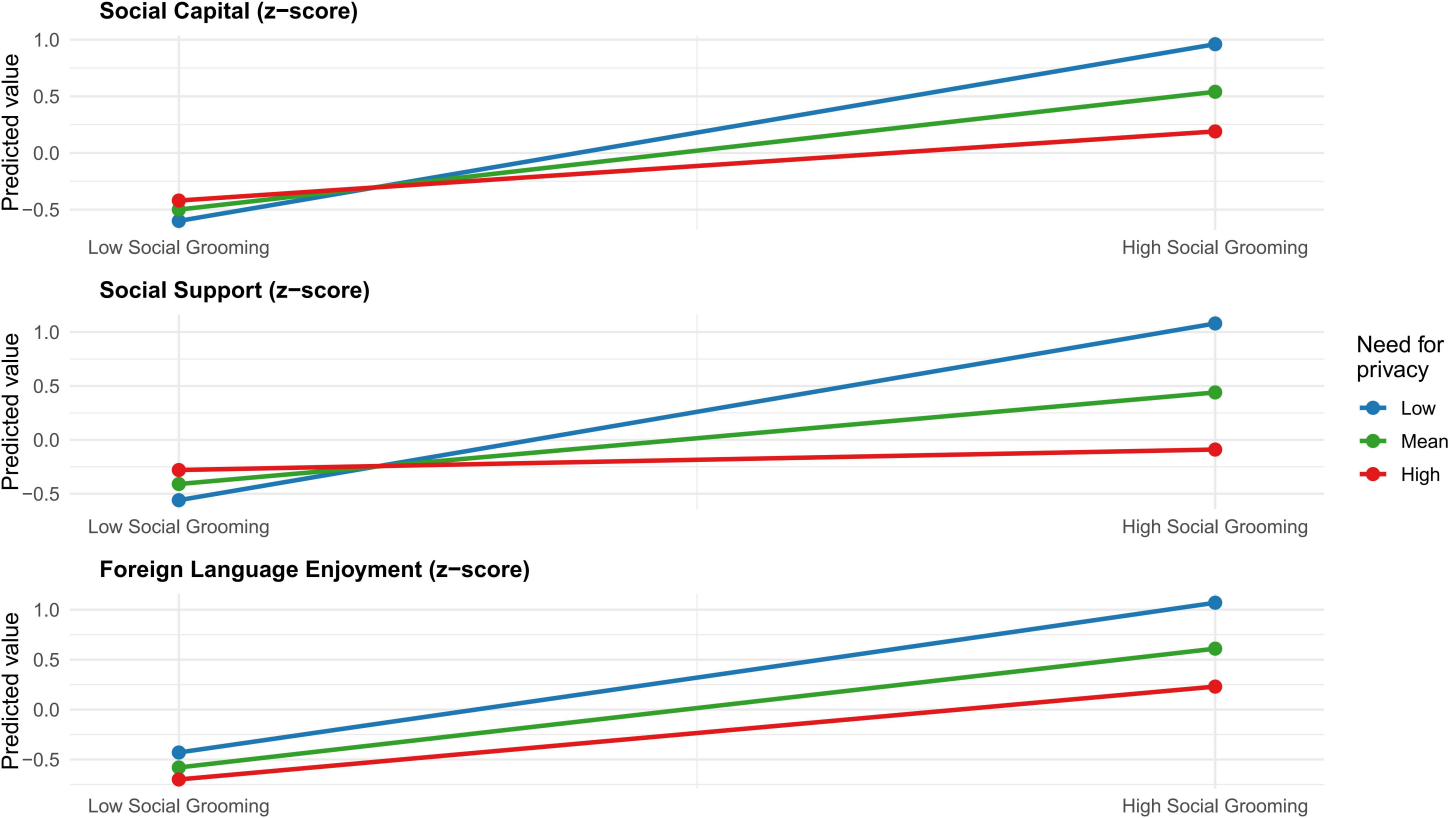
Simple slope.

Figure 2 visually supports these results. At low privacy need, intensive social grooming markedly increases social capital, social support, and foreign language enjoyment; at the median, effects remain positive but attenuated; at high privacy need, slopes are notably shallower, especially for social support.

### 4.4 Model diagnostics and robustness

Multicollinearity is within acceptable limits. The largest variance inflation factor is 4.89 (foreign language enjoyment equation), with 2.41 in the social capital equation and 3.11 in the social support equation (Table 2).

**TABLE 2:** Model diagnostics and robustness.

Criterion	Outcome
Highest VIF	Social capital: 2.41; social support: 3.11; foreign language enjoyment: 4.89
Breusch–Pagan robust *p*-values	Social capital: 0.30; social support: 0.81; foreign language enjoyment: 0.05
Robustness (HC3 vs OLS *t*-value changes)	All < 0.20
Influence diagnostics (dfbeta)	All < 0.10
Sensitivity (need for privacy split)	Maximum Δ = 0.03

Residual variance is approximately homoskedastic in the social capital and social support equations (robust Breusch–Pagan *p*-values 0.30 and 0.81, respectively) and marginal in the foreign language enjoyment equation (0.05). Because all coefficients were estimated with heteroskedasticity-consistent (type HC3) standard errors, this marginal finding does not threaten inference. Influence diagnostics (dfbeta) indicate that no observation changes any coefficient by more than 0.10, and robustness checks show that heteroskedasticity-consistent versus ordinary least squares *t*-values differ by less than 0.20 across coefficients. Together, these diagnostics support the statistical soundness of the model and the credibility of the causal estimates.

In addition to the diagnostics reported above, we conducted robustness analyses by re-estimating all models with age and gender entered as covariates. The inclusion of these demographic factors did not materially change the main conclusions: the direct effects of grooming intensity on foreign language enjoyment remained significant and positive, and the pattern of moderated mediation through social capital and social support was directionally consistent. However, several coefficients were modestly attenuated, and the sequential indirect effect (social grooming → social capital → social support → foreign language enjoyment) was no longer statistically significant. This attenuation is unsurprising given the restricted age range of participants (18–25) and the balanced gender distribution, which limits the explanatory value of these covariates. Detailed regression tables for the covariate-adjusted models are provided in the material. Therefore, the covariate analyses reinforce the robustness of the primary findings, while offering more conservative estimates of effect sizes and highlighting the need for cautious interpretation of smaller indirect pathways.

### 4.5 Sensitivity analysis

To verify that conclusions do not depend on how need for privacy is operationalized, we re-estimated the full moderated mediation model twice, using (a) informational need for privacy and (b) psychological need for privacy as the moderator. Across both re-analyses (Supplementary material), all key patterns replicated: the interaction between social grooming and need for privacy remained negative and significant on social capital and social support, and the direct path to foreign language enjoyment was again weakened by higher privacy need. Conditional direct and indirect effects at low, mean, and high levels of the moderator reproduced the same ordering observed in the main model (largest at low privacy need, smallest at high privacy need).

## 5 Discussion

This study addressed the question of how social grooming on social media influences foreign language enjoyment, and whether these effects are mediated by social ties and moderated by learners’ need for privacy Prior research on social media in language learning has produced mixed results (Derakhshan and Hasanabbasi, 2015; Pikhart and Botezat, 2021; Shamsi and Bozorgian, 2022; Shu, 2023), with some studies emphasizing motivation and engagement (Jin, 2024; Reinhardt, 2019) and others highlighting digital distraction (Cholili and Hamim, 2024), peer pressure and elevated anxiety (Shu, 2023). The present study sought to reconcile these inconsistencies by framing routine online interactions, such as posting progress updates, liking classmates’ content, and commenting on peers’ practice clips, as deliberate acts of social grooming that sustain interpersonal relationships and shape affective outcomes.

The findings demonstrate three main patterns. First, social grooming exerted a strong direct effect on foreign language enjoyment. Even after accounting for privacy need, grooming predicted substantial increases in enjoyment. Specifically, self-reported compliance logs can overstate actual engagement because of recall and social desirability, and using the same source and wave for both grooming. Enjoyment introduces common-method variance that can heighten observed associations. The 7-day window captures short-term uplift linked to task salience and social validation (e.g., heightened attention to posting and immediate peer feedback) rather than durable change. The novelty of being instructed to increase visible interaction can also induce demand characteristics effects that temporarily elevate positive affect. In addition, eligibility screening produced a relatively homogeneous sample, so range restriction can make standardized changes appear larger even when absolute changes are modest. The manipulation check and outcome survey occurred close in time, aligning perceptions of what participants did with how they felt, which can further magnify the direct link. Covariate-adjusted models that included age and gender yielded directionally consistent but attenuated effects, further suggesting that the direct pathway, while robust, is likely smaller in practical terms.

Second, social grooming also produced smaller but significant indirect effects through social capital and social support, operating both independently and sequentially. These effects formed a coherent pattern but remained modest in size, showing that social relationships play a complementary role in linking grooming to enjoyment. Consistent with the social grooming studies (Lin, 2019; Lin and Chu, 2021; Liu and Yeo, 2024; Wang and Wang, 2025), intensive language-focused grooming set in motion two convergent relational processes. First, it broadened and strengthened learners’ social capital (Mishra, 2020). Frequent small disclosures helped maintain ties and extend network connections (Dong et al., 2023). Second, the enlarged network became a conduit for emotional and instrumental help. This increased perceived social support (Venter, 2019), which is commonly observed in online peer interaction. A sequentially mediated path model showed that social capital and social support operated together (Liu and Yeo, 2024). They partly explained the association between grooming intensity and foreign-language enjoyment. However, in models adjusted for age and gender, the indirect pathways were further reduced, and the sequential route through both mediators was no longer significant. This indicates that, while statistically consistent, the indirect effects add incremental explanatory value rather than serving as the primary mechanism of influence. Their limited magnitude likely reflects the short intervention period, which constrained opportunities for deeper relationship-building, and the fact that resources such as social capital and support usually accumulate over longer timescales.

Third, learners’ need for privacy systematically weakened each of these effects. High privacy need reduced the extent to which grooming translated into social capital, social support, and ultimately, enjoyment. In this study, need for privacy is conceptualized and measured as a stable, trait-like disposition. Situational boundary-management behaviors (e.g., audience restriction, selective sharing) are described as contextual expressions of this disposition, not as the disposition itself. In other words, situational boundary strategies are not treated as separate constructs but as behaviors shaped by this disposition. This pattern reflects that individuals with stronger privacy concerns may restrict disclosure, limit reciprocal engagement, or perceive interactions as more intrusive, which reduces the effectiveness of grooming behaviors. The moderating pattern suggests that privacy preference functions as a gatekeeper: when boundary concerns are modest, grooming readily converts into capital, support, and enjoyment; when privacy need is pronounced, the same behavior is accompanied by restrictive audience management and cautious self-disclosure, curbing the flow of relational benefits. As a result, the moderating role of privacy underscores that the benefits of grooming are conditional, varying with learners’ willingness to engage openly in social exchanges.

Theoretically, these results make three contributions. First, they recast ordinary digital behaviors, posting, liking, commenting, as relational acts of social grooming, moving discussion beyond vague notions of “online engagement” toward a process-oriented account with clear antecedents and outcomes. Second, they integrate social capital theory and social support perspectives into a single sequential pathway, showing that grooming expands networks, which in turn provide emotional and instrumental resources that enrich the affective experience of learning. Third, learners’ need for privacy act as boundary conditions that regulate the benefits of grooming. This framework helps explain that the effects of social media are contingent rather than universal.

Practically, the study highlights three implications. For instructors, the focus should be on fostering purposeful grooming, interactions that invite dialogue and acknowledge peers, rather than on simply increasing the volume of online activity. For platform designers, fine-grained visibility and privacy controls are essential, enabling learners with higher privacy needs to participate without feeling overexposed. For learners themselves, the findings offer a roadmap for balancing openness and protection: carefully calibrating disclosure can transform everyday interactions into motivational support while preserving personal boundaries.

The study has several limitations that should be acknowledged. While social grooming showed a strong direct effect on enjoyment, the indirect pathways through social capital and social support were relatively modest, raising questions about their standalone practical significance. Although theoretically coherent, these smaller effects suggest that the main benefit of grooming lies in its direct influence, with interpersonal resources playing a complementary but less powerful role. Although eligibility was screened using standardized national benchmarks (CET-4 or the Gaokao English section), this approach ensured comparability but is not equivalent to administering a baseline proficiency test within the study. Future research should incorporate standardized pre-tests to better account for individual differences. In addition, the sample consisted of Mandarin-speaking Chinese university students, which limits the generalizability of the findings to other cultural and linguistic contexts. Learners navigating heritage or minority-majority language environments may experience different dynamics of privacy and support, highlighting the need for replication across more diverse populations.

Methodologically, several caveats remain. Pre-intervention measures of enjoyment, social capital, social support, and privacy need were not collected, which restricts the ability to rule out pre-existing group differences. Grooming was defined only by frequency, without assessing interaction quality, reciprocity, or affective content. Because compliance with the high- and low-grooming assignments was tracked through self-reported activity logs rather than independent verification (e.g., digital trace data), there is a risk of reporting bias. Such reliance on self-reports could potentially inflate the observed direct effects of grooming on enjoyment. Future studies should incorporate objective digital indicators to provide more accurate measures of compliance. In this study, need for privacy was measured as a trait-like disposition. Future work should also include state-level measures (e.g., experience sampling) and experimental manipulations of privacy cues to separate trait–state effects. Moreover, while grooming intensity was manipulated, privacy need was measured rather than experimentally varied, leaving causal inferences about boundary management incomplete. Future research should incorporate pre–post designs, use digital trace data, evaluate qualitative dimensions of interaction, and test privacy need experimentally or longitudinally to better capture its role in shaping the relational processes of language learning.

## 6 Conclusion

In multilingual learning environments, fostering enjoyment is critical to sustaining foreign language engagement. This study shows that purposeful social grooming on social media, through posts, likes, and comments, can enhance language enjoyment by activating interpersonal resources such as social capital and support. Crucially, these benefits are not universal. Learners with a high need for privacy experience diminished gains at each stage of this interpersonal process. By integrating Communication Privacy Management theory into the social grooming framework, the study clarifies how digital interaction supports or inhibits enjoyment in language learning. This boundary-sensitive perspective not only reconciles conflicting findings in prior research but also offers practical insight: in order to sustain heritage or foreign language use online, learners must feel in control of their visibility. These findings highlight the importance of flexible privacy design in social media platforms, especially in culturally and linguistically diverse contexts.

## Data Availability

The raw data supporting the conclusions of this article will be made available by the authors, without undue reservation.
