# Supplementary material for: Let’s post more! The impact of foreign language social grooming on social media on learners’ enjoyment: a moderated mediation model
Source: Front Psychol. 2025 Oct 8;16:1674786. doi: 10.3389/fpsyg.2025.1674786 (PMC12540393; doi:10.3389/fpsyg.2025.1674786)
Supplement: Supplementary file 1 [file Table_1.docx]

Supplementary Material

# Sensitivity analysis

Model diagnostics were stable. For **psychological privacy**, the largest variance inflation factor was **4.91** (foreign language enjoyment equation), with **3.01** (social support) and **2.26** (social capital); robust Breusch–Pagan probability values were **.32** (social capital), **.55** (social support), and **.07** (foreign language enjoyment). For **informational privacy**, the largest variance inflation factor was **4.64** (foreign language enjoyment), with **2.99** (social support) and **2.50** (social capital); robust Breusch–Pagan probability values were **.27** (social capital), **.91** (social support), and **.04** (foreign language enjoyment). Because all coefficients were estimated with heteroskedasticity-consistent (type HC3) standard errors, these marginal residual‐variance findings in the foreign language enjoyment equation do not threaten inference. Across specifications, absolute changes in the corresponding regression coefficients were **no larger than 0.04**, indicating that the dampening role of privacy concerns is robust to operationalization (informational versus psychological).

**Table 1: Conditional indirect effects**

| **Outcome and path** | **Privacy dimension** | **Low (−1 SD)** | **Mean** | **High (+1 SD)** | **Index of moderated mediation** |
| --- | --- | --- | --- | --- | --- |
| Social grooming → Social capital → Foreign language enjoyment | Informational | 0.24 (0.13–0.37) | 0.16 (0.09–0.25) | 0.09 (0.04–0.15) | −0.07 (−0.13 to −0.03) |
|  | Psychological | 0.25 (0.14–0.37) | 0.17 (0.09–0.25) | 0.09 (0.04–0.16) | −0.08 (−0.13 to −0.04) |
| Social grooming → Social support → Foreign language enjoyment | Informational | 0.23 (0.09–0.38) | 0.12 (0.05–0.20) | 0.02 (−0.02–0.06) | −0.10 (−0.18 to −0.04) |
|  | Psychological | 0.25 (0.10–0.41) | 0.13 (0.05–0.21) | 0.01 (−0.02–0.05) | −0.12 (−0.20 to −0.05) |
| Social grooming → Social capital → Social support → Foreign language enjoyment | Informational | 0.04 (0.01–0.08) | 0.03 (0.01–0.06) | 0.02 (0.00–0.03) | −0.01 (−0.03 to 0.00) |
|  | Psychological | 0.04 (0.01–0.08) | 0.03 (0.01–0.06) | 0.01 (0.00–0.03) | −0.01 (−0.03 to 0.00) |

Note: percentile bootstrap, 5,000 samples; effects with 95% confidence intervals

**Table 2: Conditional direct effects of social grooming on foreign language enjoyment**

| **Privacy dimension** | **Low (−1 SD)** | **Mean** | **High (+1 SD)** |
| --- | --- | --- | --- |
| Informational | 1.46 (1.24–1.67) | 1.16 (1.02–1.31) | 0.87 (0.71–1.02) |
| Psychological | 1.47 (1.25–1.69) | 1.19 (1.05–1.33) | 0.90 (0.75–1.05) |

Note: **with HC3 standard errors and 95% confidence intervals**

**Table 3: Interaction (product-term) estimates for reference**

| Outcome equation | Informational privacy (GI × ZNP_I) | Psychological privacy (GI × ZNP_P) |
| --- | --- | --- |
| Social capital | −0.47 (SE = 0.09), p < .001 | −0.50 (SE = 0.10), p < .001 |
| Social support | −0.69 (SE = 0.08), p < .001 | −0.78 (SE = 0.07), p < .001 |
| Foreign language enjoyment | −0.29 (SE = 0.06), p < .001 | −0.28 (SE = 0.06), p < .001 |

**Table 4: Diagnostics summary**

| Metric | Informational privacy | Psychological privacy |
| --- | --- | --- |
| Highest variance inflation factor | Foreign language enjoyment = 4.64; Social support = 2.99; Social capital = 2.50 | Foreign language enjoyment = 4.91; Social support = 3.01; Social capital = 2.26 |
| Robust Breusch–Pagan probability values | Social capital = .27; Social support = .91; Foreign language enjoyment = .04 | Social capital = .32; Social support = .55; Foreign language enjoyment = .07 |

Notes. Moderator levels correspond to the 16th (low), 50th (mean), and 84th (high) percentiles reported by PROCESS. Confidence intervals for indirect effects are percentile bootstrap intervals based on 5,000 samples. All continuous variables are standardized (z-scores); the independent variable is dichotomous (0 = low social grooming, 1 = high social grooming). All coefficients are estimated with heteroskedasticity-consistent (type HC3) standard errors.

**Table 5: Covariance Analysis**

| **Outcome Variable** | **Predictor** | **b** | **SE** | **t** | **p** | **90% CI LL** | **90% CI UL** |
| --- | --- | --- | --- | --- | --- | --- | --- |
| Social Capital | Grooming (GI) | 1.05 | 0.09 | 11.3 | 0 | 0.9 | 1.2 |
|  | Age | 0.02 | 0.02 | 0.95 | 0.34 | -0.01 | 0.05 |
|  | Gender (male = 1) | -0.08 | 0.09 | -0.9 | 0.37 | -0.24 | 0.07 |
| Social Support | Grooming (GI) | 0.87 | 0.09 | 9.53 | 0 | 0.72 | 1.02 |
|  | Age | 0 | 0.02 | 0.13 | 0.89 | -0.03 | 0.03 |
|  | Gender (male = 1) | -0.06 | 0.08 | -0.74 | 0.46 | -0.19 | 0.07 |
| FLE | Grooming (GI) | 1.19 | 0.07 | 16.55 | 0 | 1.07 | 1.31 |
|  | Social Capital | 0.16 | 0.04 | 4.37 | 0 | 0.1 | 0.22 |
|  | Social Support | 0.14 | 0.05 | 2.99 | 0.003 | 0.06 | 0.21 |
|  | Age | -0.02 | 0.01 | -1.46 | 0.15 | -0.04 | 0 |
|  | Gender (male = 1) | 0 | 0.05 | -0.05 | 0.96 | -0.09 | 0.09 |
